# The cell-free DNA methylome captures distinctions between localized and metastatic prostate tumors

**DOI:** 10.1038/s41467-022-34012-2

**Published:** 2022-10-29

**Authors:** Sujun Chen, Jessica Petricca, Wenbin Ye, Jiansheng Guan, Yong Zeng, Nicholas Cheng, Linsey Gong, Shu Yi Shen, Junjie T. Hua, Megan Crumbaker, Michael Fraser, Stanley Liu, Scott V. Bratman, Theodorus van der Kwast, Trevor Pugh, Anthony M. Joshua, Daniel D. De Carvalho, Kim N. Chi, Philip Awadalla, Guoli Ji, Felix Feng, Alexander W. Wyatt, Housheng Hansen He

**Affiliations:** 1 Princess Margaret Cancer Center, University Health Network, Toronto, ON Canada; 2 Department of Medical Biophysics, University of Toronto, Toronto, ON Canada; 3 West China School of Public Health, West China Fourth Hospital, and State Key Laboratory of Biotherapy, Sichuan University, Chengdu, China; 4 Department of Automation, Xiamen University, Xiamen, Fujian China; 5 National Institute for Data Science in Health and Medicine, Xiamen University, Xiamen, Fujian China; 6 College of Electrical Engineering and Automation, Xiamen University of Technology, Fujian, Xiamen China; 7 Department of Radiation Oncology, University of California, San Francisco, CA USA; 8 UCSF Helen Diller Family Comprehensive Cancer Center, San Francisco, CA USA; 9 Department of Medical Oncology, Kinghorn Cancer Centre, St Vincent’s Hospital, Sydney, NSW Australia; 10 Sunnybrook Research Institute, Sunnybrook Health Sciences Centre, Toronto, ON Canada; 11 Department of Radiation Oncology, Sunnybrook Odette Cancer Centre, Toronto, ON Canada; 12 Department of Pharmacology & Toxicology, University of Toronto, Toronto, ON Canada; 13 British Columbia Cancer Agency, Vancouver Centre, Vancouver, BC Canada; 14 Ontario Institute for Cancer Research, Toronto, ON Canada; 15 Division of Hematology and Oncology, Department of Medicine, University of California San Francisco, San Francisco, CA USA; 16 Department of Urology, University of California San Francisco, San Francisco, CA USA; 17 Vancouver Prostate Centre, Department of Urologic Sciences, University of British Columbia, Vancouver, BC Canada

**Keywords:** Cancer genomics, Prostate cancer

## Abstract

Metastatic prostate cancer remains a major clinical challenge and metastatic lesions are highly heterogeneous and difficult to biopsy. Liquid biopsy provides opportunities to gain insights into the underlying biology. Here, using the highly sensitive enrichment-based sequencing technology, we provide analysis of 60 and 175 plasma DNA methylomes from patients with localized and metastatic prostate cancer, respectively. We show that the cell-free DNA methylome can capture variations beyond the tumor. A global hypermethylation in metastatic samples is observed, coupled with hypomethylation in the pericentromeric regions. Hypermethylation at the promoter of a glucocorticoid receptor gene *NR3C1* is associated with a decreased immune signature. The cell-free DNA methylome is reflective of clinical outcomes and can distinguish different disease types with 0.989 prediction accuracy. Finally, we show the ability of predicting copy number alterations from the data, providing opportunities for joint genetic and epigenetic analysis on limited biological samples.

## Abstract (web summary)

Metastatic prostate cancer can be difficult to biopsy and characterise. Here, the authors use cell-free DNA methylation analysis to illustrate changes in hypermethylation in metastatic disease.

## Introduction

Prostate cancer (PCa) poses a significant clinical burden as the second most common malignancy in men and the third most common cause of cancer-related death worldwide^1^. While most localized PCa can be cured, the 5-year survival rate for patients presenting with metastatic disease is as low as 30%^2^. In recent years, there has been an increased incidence rate for metastatic cases^3^. Androgen deprivation therapy (ADT) treatment is the standard of care for patients with advanced or metastatic disease. However, despite initial effectiveness, most patients progress to metastatic castration-resistant prostate cancer (mCRPC) shortly, and eventually, almost all will die from it. CRPC cells grow independently of testosterone stimulation by developing mechanisms to constitutively activate the androgen signaling pathway. Development and application of the more potent, second-generation androgen signaling inhibitors (ASI) like enzalutamide and abiraterone acetate were able to provide additional survival benefits for CRPC patients and are increasingly applied in the earlier lines of treatment for advanced disease^4–8^. However, drug resistance will ultimately develop, and these agents fail to suppress tumor progression. There is thus an urgent need to improve our understanding and treatment of mCRPC.

Biopsy for mCRPC lesions is challenging, even more so when trying to obtain sufficient materials for molecular analysis^9^. Analysis of circulating tumor DNA (ctDNA) in liquid biopsies has shown potential as a minimally invasive and accurate disease monitoring tool^10,11^. ctDNA refers to the component of total cell-free DNA (cfDNA) that is derived from tumor cells circulating in the bloodstream. It has been demonstrated that quantification of ctDNA can provide information on tumor burden, metastasis, and treatment response^12^. Of interest, in mCRPC, ctDNA has been shown to reflect the genomic profiles of tumor or metastatic lesions, and epigenetic characteristics such as methylation status can reflect tumor burden and subtypes^13–15^. Despite these advantages, genome-wide cfDNA methylome profiles of mCRPC in large-scale clinical cohorts are still lacking. Recently, the development of cell-free methylated DNA immunoprecipitation coupled with next-generation sequencing (cfMeDIP-seq) presents an effective approach for the analysis of cfDNA methylomes^16^. This approach allows for sensitive detection of ctDNA from minute quantities of cfDNA and is more cost-effective compared to genome-wide bisulfite-based methods^17–19^.

Here, we analyze the cell-free methylome of 60 localized and 175 metastatic samples with the cfMeDIP-seq technology. The global methylome captures variations reflective of the heterogeneous disease biology. We further show that the cell-free methylome can distinguish different disease status with high accuracy, highlighting its potential as a minimally invasive strategy for disease monitoring and prognostication.

## Results

### A genome-wide analysis of plasma DNA methylome in localized and metastatic PCa

**Fig. 1:**
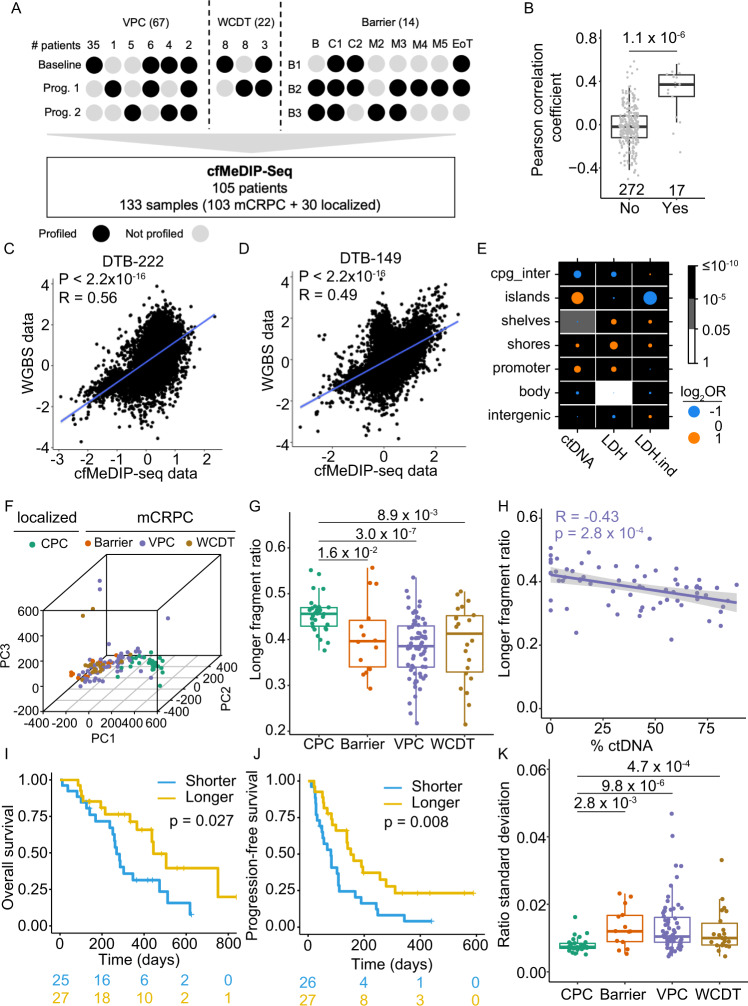
cfMeDIP data capture cell-free DNA methylation and fragmentation changes in localized vs. mCRPC patient plasma samples. **A** Sampling schematics for the three mCRPC cohorts. Numbers in parentheses indicate the total number of samples in given cohorts. For the Vancouver Prostate Cancer (VPC) and West Coast Dream Team (WCDT) cohorts, each column represents a sampling strategy, and the number above shows the number of patients subjected to the indicated sampling strategy. For the Barrier cohort, each column represents a different time point and each row represents a patient. B baseline, C1/C2 treatment cycle 1/2; M2/3/4/5, 2/3/4/5 months post C2; EoT, end of the trial. **B** Pairwise sample correlation between tissue WGBS and cfMeDIP data in the WCDT cohort, matched samples collected from the same patient were compared with the others. *P* value = 1.1 × 10^−6^ (two-sided Mann–Whitney *U* test). Box plots represent median values and 0.25 and 0.75 quantiles. Whiskers represent 1.5× interquartile range (IQR). *X* = 272 and 17 independent observations for the “No (matched)” and “Yes (unmatched)” groups, respectively. Scatterplot showing the top 10,000 most variable bins in tissue WGBS and cfMeDIP data for patient DBT-222 C) and DBT-149 (**D**). **E** Enrichment in different genomic locations for bins that are well explained by %ctDNA, LDH, and LDH independent of %ctDNA. (Delta) *R* squared of 0.2 is used as a cutoff to filter for regions that are well explained by the indicated variable. Fisher’s exact test was used to calculate *p* value and odds ratio. **F** Three-dimensional representation of samples according to the principal component analysis (PCA) for the four cohorts using the top 10,000 most variable bins. **G** Ratio of longer cfDNA fragments across the cohorts. Two-sided Mann–Whitney U test was used to calculate pairwise *p* values between localized samples from the CPC and mCRPC samples from the Barrier, VPC, WCDT cohorts, respectively. Color code is the same as used in **F**. Box plots represent median values and 0.25 and 0.75 quantiles. Whiskers represent 1.5× interquartile range (IQR). *X* = 30, 14, 67, and 22 independent experiments for the CPC, Barrier, VPC, and WCDT cohorts, respectively. **H** Pearson correlation of longer fragment ratio and ctDNA fraction (%ctDNA) in mCRPC samples from the VPC cohorts. *P* value was calculated using a two-sided *t* test. Purple line represents a fitted linear model of the data and shading around the fitted line represents 0.95 confidence interval (CI). Association of samples with shorter or longer fragment sizes with overall survival (**I**) and progression-free survival (**J**). Median value of the longer fragment ratio (0.3933) was used to dichotomize samples into two groups. **K** Distribution of the standard deviation of fragment ratio within a sample. Two-sided Mann–Whitney U test was used to calculate pairwise *P* values between localized samples from the CPC and mCRPC samples from the Barrier, VPC, WCDT cohorts, respectively. Color code same as used in **F**. Box plots represent median values and 0.25 and 0.75 quantiles. Whiskers represent 1.5× IQR. X = 30, 14, 67, and 22 independent experiments for the CPC, Barrier, VPC, and WCDT cohorts, respectively. Source data for 1**B**, 1**F**–**H**, and 1**K** are provided as a Source Data file.

To gain a deeper understanding of the cfDNA methylation profile changes during PCa progression, we curated a total of 133 plasma samples, including 30 and 103 plasma samples from patients with primary and mCRPC, respectively (Fig. 1A, Supplementary Data 1). The localized tumor plasma samples were collected as part of the Canadian Prostate Cancer Genome Network (CPC-GENE, CPC for short) project^20^, while the mCRPC cases were sampled from three well-curated cohorts of metastatic PCa (Fig. 1A): (1) 67 plasma samples from a randomized phase II clinical trial (NCT02125357) comparing the sequential use of abiraterone and enzalutamide on first-line mCRPC^11^ at Vancouver Prostate Centre (VPC). (2) 14 plasma samples were collected from three patients over the course of an enzalutamide treatment trial (Barrier). (3) 22 plasma samples from patients enrolled in the West Coast Prostate Cancer Dream Team (WCDT) study^21^. For the VPC and WCDT cohorts, we profiled 47 and 11 samples collected at the time of enrollment (baseline); 30 and 11 samples upon PSA progression after targeted AR inhibition treatment (progression), respectively (Fig. 1A). Barrier cohort consisted of samples collected at baseline and along the treatment course (Fig. 1A, Supplementary Data 1). Together, the datasets here form a comprehensive representation of PCa, particularly for metastatic castration-resistant lesions. Furthermore, mCRPC samples from the VPC cohort has cfDNA sequencing profiled, and the CPC (localized) and WCDT (mCRPC) cohorts have multi-omics sequencing data available for matched tissues in previous studies (Supplementary Data 1)^11,21–25^, providing unique opportunities for integrative analysis.

Methylated DNA fragments from all plasma samples were precipitated and subjected to paired-end DNA sequencing as described before^26^ (Methods). We obtained a median of 63 million reads, covering over 60% of the genome, and showed enrichment of CpGs (Methods) between 1.59 and 2.19 fold (Supplementary Data 1). We used the previously described binning strategy^16^ to quantify the data in 300 bp non-overlapping bins and reduced our analysis to the 337,420 non-low bins (Methods, Supplementary Data 2). In general, there is a higher correlation between samples collected from the same patients at different timepoints (baseline or progression) (Supplementary Fig. 1A), suggesting higher inter-patient variations. We further compared the cell-free methylome with previous tissue whole-genome bisulfite sequencing (WGBS) profiles^21^ from the WCDT cohort using the top 10,000 most variable bins. Despite differences in sequencing technologies, a significantly higher correlation was observed between the matched tissue and cell-free methylomes, with the median Pearson’s *r* for matched samples being 0.37 compared to −0.02 for unmatched samples (Fig. 1B–D).

### cfMeDIP data captures variations from tumor and TME

To understand the source of data variations, we examined major clinical covariates by fitting linear regression models for individual bins using the 67 mCRPC samples from the VPC cohort (Supplementary Fig. 1B–E). Adjusted R squared was used to measure the proportion of variation explained by different covariates for each non-low bin. For less than one-fifth (19.63%) of the bins, variations in methylation signal showed a weak to moderate correlation (adjusted *R* squared >0.2) with ctDNA fraction (%ctDNA)^11^, and for only 3.45% of the regions %ctDNA has a moderate to high correlation (adjusted *R* squared >0.5) (Supplementary Fig. 1B). This is different from previous targeted methylation analysis on plasma DNA for mCRPC samples, where tumor fraction was identified as the major determinant of variation^15^. The previous analysis focused on the CpG sites covered by the Roche probes and left a large proportion of the methylome uncharacterized (Supplementary Fig. 1F): 75.4% of the non-low bins in our study were not covered. We reasoned that the unbiased cfMeDIP strategy was able to provide genome-wide methylome analysis reflective of a more comprehensive source of variation.

We next investigated other available clinical factors, including age, lactate dehydrogenase (LDH) and alkaline phosphatase (ALP) levels. Serum LDH and ALP levels measure groups of enzymes that catalyze glycolysis and phosphate esters, respectively. LDH levels are associated with the outcomes in mCRPC and are related to visceral diseases such as liver metastasis^27–29^. Bone-specific ALP is expressed on the surface of osteoblasts and is commonly upregulated in cancers originating or spreading to the bone^30^. Indeed, while age barely contributes, ALP level explains the variations to a weak to moderate degree for 2.41% of the bins and is a moderate to high contributor for a smaller fraction (0.3%) (Supplementary Fig. 1C, D). Interestingly, more bins (23.77%) are explained to a weak to moderate degree by LDH level than %ctDNA (19.63%) (Supplementary Fig. 1B, E), with 17.56% of the bins explained by LDH independent of %ctDNA variation (Supplementary Fig. 1G). These bins distributed differently across the genome: bins explained to a weak to moderate degree by %ctDNA are enriched in CpG islands and promoter regions while depleted in shelves (cpg_inter) and intergenic regions (Fig. 1E). Meanwhile a sharp contrast was observed for bins explained to a weak to moderate degree by LDH independently (Fig. 1E). Together, the amount of variation explained by ALP and LDH, but not %ctDNA, is potentially reflective of variations related to changes in the tumor microenvironment (TME).

### cfMeDIP data capture fragmentation profile changes in the cfDNA

We next extended the analysis to include all four cohorts. The global methylation pattern was able to provide a general separation between the localized and metastatic data (Fig. 1F), with several mCRPC samples having low to undetectable %ctDNA clustered closer to the primary cancer cohort (Supplementary Fig. 1H, I). Previous studies reported that tumor-derived cfDNA has a shorter length compared to healthy controls^31,32^, we thus analyzed the cfDNA fragment size in the four cohorts. Significant shorter fragment size was observed in mCRPC samples compared to that of localized samples (Fig. 1G). In addition, the fragment size is significantly negatively associated (Pearson’s *r* = −0.43, *p* value = 2.8 × 10^−4^) with %ctDNA in mCRPC samples from the VPC cohort (Fig. 1H). Together, these suggest a higher %ctDNA in the metastasis compared to localized patient blood, corroborating previous observations^31^. While previous studies of total cfDNA showed significant enrichment of fragments below 150 bp in patient samples^33–35^, size distribution difference in our data mainly occurred longer than 150 bp (Supplementary Fig. 1J). We reasoned that methylated ctDNA could have different fragment lengths, and differences in experimental approaches may also contribute to such disparity. Nevertheless, our analysis showed that cfMeDIP-seq can capture fragment length difference qualitatively. As expected, the estimated fragment size distribution is significantly associated with both overall and progression-free survival (PFS, Fig. 1I, J). Besides the length difference, cancers are shown to have more variation in their cfDNA length^36^. Indeed, when we examine the fragmentation profile in 5-Mb bins across the genome (Methods), a higher standard deviation was observed in the metastatic cohorts (Fig. 1K). Differences in fragment length and fragmentation can further distinguish our localized samples from the healthy controls (HC) cfMeDIP-seq profiles reported previously^32^ (Supplementary Fig. 1K, L).

### Cell-free 5mC profiling reveals widespread hyper-methylation in metastatic samples

**Fig. 2:**
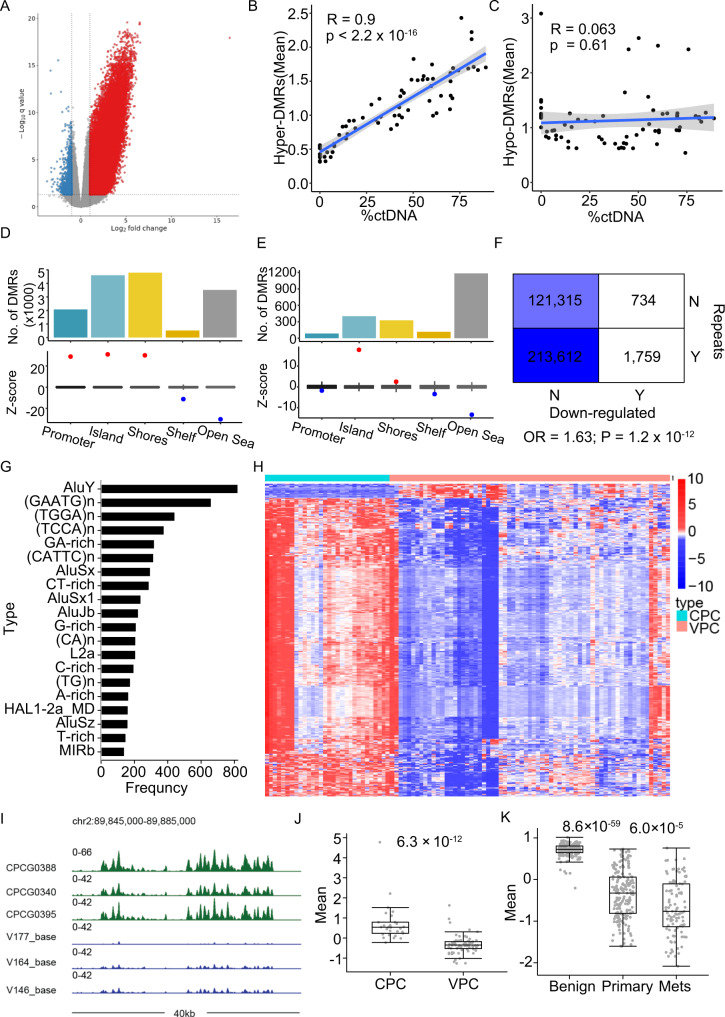
cfMeDIP reveals widespread hypermethylation and preferential repeat hypomethylation in metastatic samples. **A** Volcano plot of differentially methylated regions (DMRs) was identified comparing metastatic and localized prostate cancer. A total of 2493 and 19,048 hypo and hyper DMRs were identified, respectively. Pearson correlation between the mean methylation levels of hyper (**B**) and hypo (**C**) DMRs and %ctDNA. *P* value was calculated using *t* test. Blue line represents a fitted linear model of the data and shading around the fitted line represents 0.95 CI. Genomic distribution of hyper- (**D**) and hypo- (**E**) DMRs. **F** Contingency table showing the distribution of repeat and the hypo-DMRs. Two-sided fisher’s exact test was used to calculate *p* value and odds ratio (OR). **G** Frequency of repeat types overlapped with downregulated peaks. **H** Differentially methylated peaks located within the 1 Mb regions flanking the centromere. CPC Canadian Prostate Cancer Genome Network cohort. **I** Example showing signal distribution around the pericentromeric region in chromosome 2. The *Y* axis showed the normalized signal per million reads (SPMR) from MACS (v2.2.5). Mean methylation levels for differential peaks shown in **H** for cfMeDIP-seq (**J**) and WGBS (**K**) data. Box plots represent median values and 0.25 and 0.75 quantiles. Whiskers represent 1.5× IQR. *X* = 30, and 67 independent experiments for the CPC and VPC cohorts, respectively in **J**. *X* = 194, 194, and 100 independent experiments for the Benign, Primary, and Mets groups, respectively, in **K**. Source data for F2B-C and 2J-K are provided as a Source Data file.

We next compared the cell-free methylation profiles between the CPC (localized) and VPC (metastatic) samples with age control (Methods). Widespread hypermethylation in metastatic tumors was observed (Fig. 2A) and we detected 7.6 times (19,048 hyper vs. 2493 hypo) more differentially methylated regions (DMRs) with increased methylation than decreased. Meanwhile, previously reported hypomethylation sites in patient tumors showed consistent lower methylation in the metastatic samples^37^, corroborating the validity of our analysis (Supplementary Fig. 2A). Global methylation on promoters of tumor suppressor genes showed significantly higher methylation in mCRPCs (Supplementary Fig. 2B), whereas only moderate differences were observed for oncogenes^38,39^ (Supplementary Fig. 2C). Indeed, DNA methylation is observed as a common mechanism for transcriptional regulation for tumor suppressors but not protooncogenes^40^. The detected DMRs also showed consistent deregulation in the Barrier and WCDT cohorts (Supplementary Fig. 2D), highlighting the robustness of the detected methylation changes. These robust DMRs can stratify patients into different risk groups: A ratio score dividing hyper-DMRs by hypo-DMRs is significantly associated with both overall and PFS (Supplementary Fig. 2E, F).

In contrast to the significant positive correlation between hyper-DMRs and %ctDNA in the VPC cohorts (Fig. 2B), hypo-DMRs show no correlation (Fig. 2C). Both hyper- and hypo- DMRs are enriched in CpG islands and shores but depleted in shelf and open sea regions (Fig. 2D, E), with the former, also enriched in gene promoters. Genome occupancy of important transcription factors (TF) shows differential enrichment patterns in hyper- and hypo- DMRs (Supplementary Fig. 2G, H). Specifically, top hits enriched in hyper-DMRs are transcriptional suppressive factors like SUZ12 and EZH2, while for hypo-DMRs, activating factors like TRIM24 and CREB1 are the most enriched^41,42^. AR binding is enriched in both sets of DMRs, consistent with the recognition of its dual roles in transcriptional regulation^43,44^.

### Pericentromeric regions are preferentially hypo-methylated in metastatic samples

While hyper-DMRs are enriched in regulatory regions like the promoters, hypo-DMRs are not (Fig. 2D, E). Upon further investigation, we found hypo-DMRs to be specifically enriched in the repeat regions (Fig. 2F). For better characterization of the repeat signal, we utilized a peak calling strategy to quantify the methylation signal (Methods, Supplementary Data 2). Among the most enriched repeat types in hypomethylated peaks is GAATGn, the classic satellite DNA frequently found in pericentromeric regions^45^ (Fig. 2G). Indeed, differentially methylated peaks within the pericentromeric regions (1 Mb around the annotated centromere gaps) show considerably reduced signals in metastatic samples (Fig. 2H–J). We compared tissue WGBS data from two previous studies and observed a similar pattern^21,46^ (Fig. 2K, Supplementary Fig. 2I). In addition, a more noticeable reduction between the benign tissue and the primary tumor samples was observed, suggesting progressive loss of methylation for pericentromeric regions along the PCa development trajectory.

### Methylation level at *NR3C1* promoter associates with differential disease outcome

**Fig. 3:**
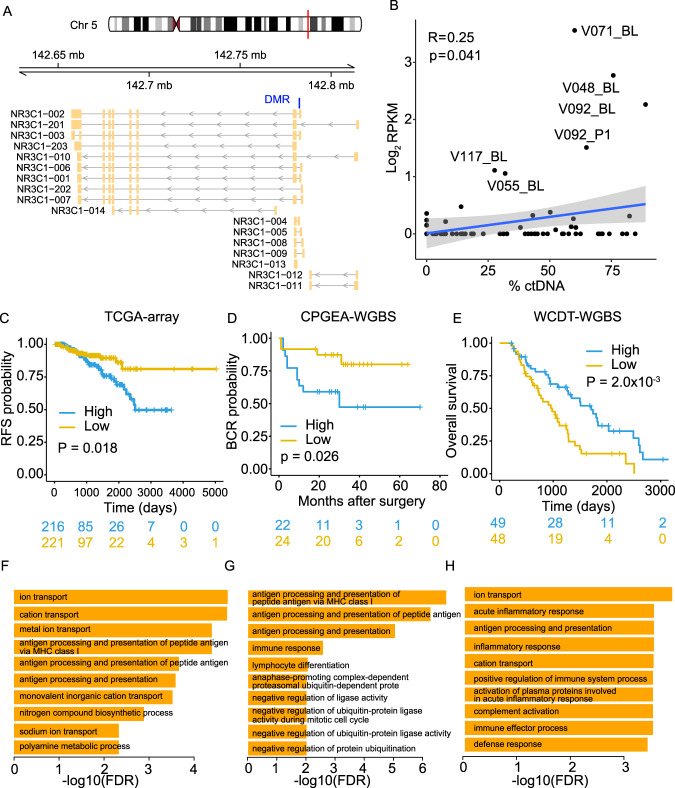
Differential methylation in GR gene associates its altered role in mCRPC. **A** Schematics illustrating GR gene (NR2C1) and its different isoforms. Blue bar indicates the identified outlier DMR. **B** Pearson correlation between GR site methylation level and %ctDNA in VPC cohort. *P* value was calculated using two-sided *t* test. Blue line represents a fitted linear model of the data and shading around the fitted line represents 0.95 CI. Association between GR site methylation and disease outcome in the Cancer Genome Atlas (TCGA) (**C**), the Chinese Prostate Cancer Genome and Epigenome Atlas (CPGEA) (**D**) and WCDT (**E**) cohorts. Logrank test was used to calculate *p* values. *X* = 216 and 221, 22 and 24, 49, and 48 for high and low-risk groups for **C**–**E**, respectively. Gene ontology (GO) analysis shows the enrichment of Biological Process (BP) for genes downregulated in high GR-DMR methylation groups in TCGA (**F**), CPGEA (**G**), and WCDT (**H**) cohorts. Source data for F3B are provided as a Source Data file.

An outlier DMR showing the highest fold change (Fig. 2A) is located in the promoter region of the gene *NR3C1* (Fig. 3A), which encodes glucocorticoid receptor (GR). The methylation level at this site (referred to as GR-DMR hereafter) shows a borderline positive correlation with %ctDNA (Fig. 3B), and the high fold change is likely driven by the few samples with exceptionally high methylation in the VPC cohort. We examined cfMeDIP-seq data of the isolated peripheral blood leukocytes from 20 healthy donors in a recent study and no signal was detected in this site^32^, suggesting cancer-specific methylation on GR-DMR. Higher methylation level at GR-DMR in localized tumors from the Cancer Genome Atlas (TCGA), the Chinese Prostate Cancer Genome and Epigenome Atlas (CPGEA) and the CPC cohorts^22,46,47^ are associated with worse outcomes (Fig. 3C, D, Supplementary Fig. 3A), while a reverse association was observed in the metastatic cohort (Fig. 3E). Associations with survival are not significant in the VPC (mCRPC) cfMeDIP-seq data (Supplementary Fig. 3B, C), likely due to small sample size. Expression levels of the GR gene only showed a moderate negative correlation with methylation levels at GR-DMR (Supplementary Fig. 3D–G). Moreover, direct GR RNA abundance showed no significant survival association except in the CPGEA cohort (Supplementary Fig. 3H–K), suggesting the existence of different regulatory mechanisms of GR expression.

To understand how the methylation changes at GR-DMR affect disease outcome, we performed differential gene expression analysis comparing samples with high and low GR-DMR methylation levels. Considering the relatively low number of samples with high GR-DMR methylation in the localized CPC cohort (Supplementary Fig. F3E), we focused on the remaining three datasets (Supplementary Fig. 3L–N). Enrichment analysis showed that downregulated genes in the hypermethylation group are significantly associated with immune-related terms, including “antigen processing and presentation” (Fig. 3F–H). Such enrichment is observed in both the localized and mCRPC cohorts, suggesting potential immune regulatory roles related to GR-DMR methylation. Top terms enriched in the upregulated genes in the CPGEA cohort are all cell cycle-related (Supplementary Fig. 3O**)**. Although not significant, these cell cycle-related genes show the trend of upregulation in the TCGA cohort (Supplementary Fig. 3P), consistent with the worse outcome associated with high GR-DMR methylation observed in primary tumors (Supplementary Fig. 3C, D). In contrast, such upregulation is not observed in the WCDT cohort (Supplementary Fig. 3P), suggesting a trend of switching from cell cycle regulatory roles in primary tumors to a dominant immune regulation in metastatic cases for the GR-DMR methylation.

### Cell-free DNA methylome distinguish metastatic from localized samples with high accuracy

**Fig. 4:**
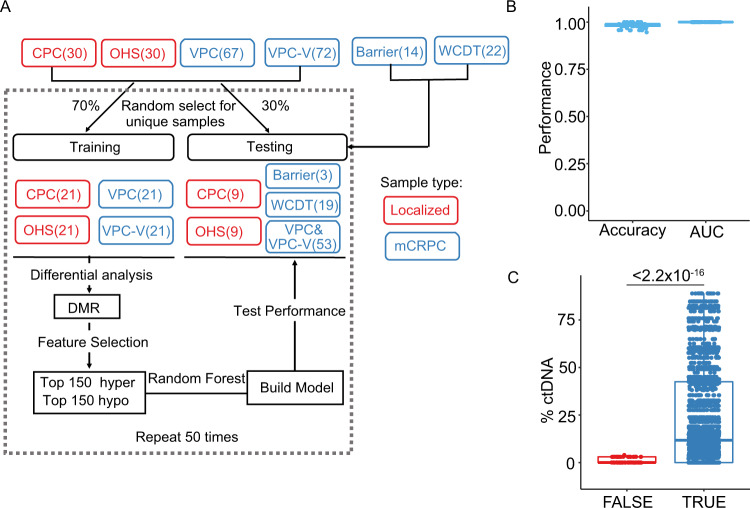
Methylation profiles distinguish localized from metastatic samples with high accuracy. **A** Workflow of building a random forest predictor using methylation profiles. **B** Prediction accuracy and area under the receiver operating characteristics (AUROC) for methylation profile-based predictor on testing datasets. VPC-V validation samples from the VPC cohort, OHS the Ontario Health Study cohort, DMR differentially methylated region. **C** %ctDNA distribution in samples that are correctly or misclassified for the metastatic samples. Results from the 50 repeats were pooled for visualization. Two-sided Mann–Whitney U test was used to calculate pairwise *P* values. Box plots represent median values and 0.25 and 0.75 quantiles. Whiskers represent 1.5× IQR. X = 69 and 2656 test results pooled from 50 times of repetition for FALSE and TRUE groups, respectively. Source data for F4B-C are provided as a Source Data file.

We next sought to create a machine-learning predictor distinguishing localized and metastatic tumors using the methylation profiles. To increase the sample size, we sequenced an additional 72 samples from the VPC cohort (VPC-V), and 30 samples from patients with localized tumor samples from the Ontario Health Study (OHS) cohort (Fig. 4A, Supplementary Fig. 4A). To avoid potential bias caused by the imbalanced classification in our samples, we randomly select equal numbers (21 for each) of localized and metastatic samples from the respective cohorts and use them as a training set (Fig. 4A). Feature selection was then performed on the training set: differential methylation analysis was performed and the top 150 hyper- and hypo- DMRs were selected. A random forest classifier was then built using the selected features and evaluated on the remaining dataset (testing set). This process was repeated 50 times and the performances were summarized (Fig. 4A–C, Supplementary Fig. 4B).

The predictor yielded a median area under the curve value of 1, with a very high median accuracy of 0.989 on the testing datasets (Fig. 4B, Supplementary Fig. 4B, C). All the localized samples were correctly classified, with only 10 out of the 175 metastatic samples ever misclassified across the 50 repeats (Fig. 4C). It is worth noting most samples with very low %ctDNA (<2%) were correctly classified, highlighting the sensitivity of DNA methylation in detecting samples with lower load of genetic alterations (Fig. 4C). We further applied our predictors to the 20 healthy controls from the previous study^32^. Significant probability distribution difference from the localized samples was observed (Supplementary Fig. 4C), suggesting the potential of discriminating between early-stage disease and healthy controls using cfMeDIP-seq data.

### Cell-free DNA methylome can predict large-scale genetic variations

**Fig. 5:**
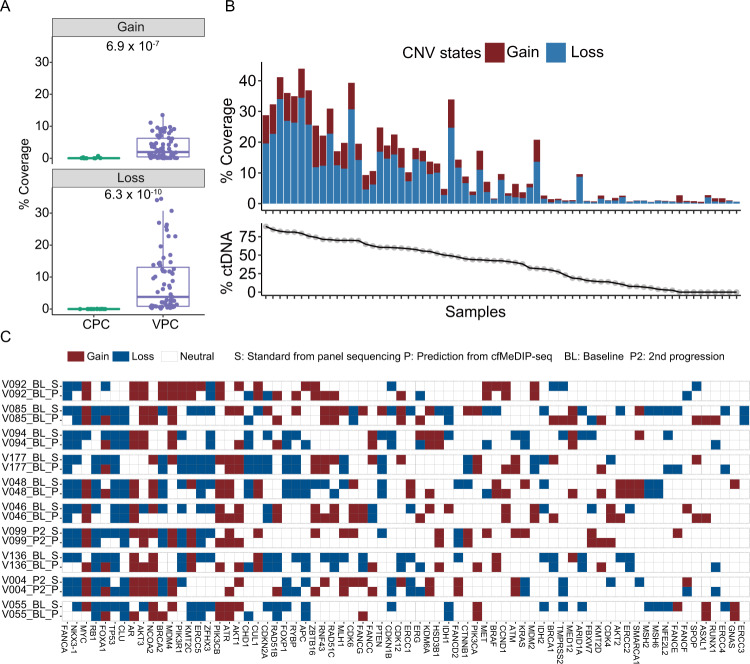
Prediction of sample CNA using cfDNA methylome. **A** Predicted CNA coverage in localized samples from the CPC and mCRPC samples from the VPC cohorts. Two-sided Mann-Whitney U test was used to calculate pairwise *P* values. Box plots represent median values and 0.25 and 0.75 quantiles. Whiskers represent 1.5× IQR. *X* = 30, and 67 independent experiments for the CPC and VPC cohorts, respectively. **B** Correlation between the predicted CNA coverage and %ctDNA in mCRPC samples from the VPC cohorts. **C** Comparison of gene CNA between standard status obtained from previous panel sequencing^11^ and predicted results from cfMeDIP-seq data for the top 10 samples with the highest CNA coverage. Source data for F5A-B are provided as a Source Data file.

Sequencing information obtained from cfMeDIP-seq data can reflect genetic changes and enable copy number alteration (CNA) analysis. Similar to RNA-seq data, enrichment-based cfMeDIP data also has uneven coverage caused by different methylation levels when used for CNA analysis. We thus adapted the CNA inference tool for RNA-seq to the analysis of cfMeDIP-seq data^48^ (Methods). Overall, CNA coverage is significantly higher in VPC (Fig. 5A), consistent with the notion of higher %ctDNA associated with metastatic samples. Indeed, CNA coverage showed a significant positive correlation with %ctDNA in the VPC cohorts (Fig. 5B, Supplementary Fig. 5A). We next examined the CNA changes in detail for individual genes assayed by panel sequencing from a previous study^11^ and high concordance was observed (Fig. 5C, Supplementary Fig. 5B). In regions that were misclassified as CNA neutral, a significantly lower degree of CNA was observed (Supplementary Fig. 5C). Very few depletions (9) were misclassified as amplifications and even fewer amplifications (3) were misclassified as depletions. Majority of the CNA neutral regions were correctly predicted, with 4% misclassification (154/3835) (Supplementary Fig. 5C). Together, these resulted in an overall accuracy of 0.86 for CNA prediction (Table 1). When considering only regions with CNA change, a 0.975 accuracy was achieved (Supplementary Fig. 5D). We then extended the analysis to the VPC-V. Only a moderate correlation between the predicted CNA coverage and %ctDNA (Supplementary Fig. 5E) was observed, likely due to the reduced sensitivity caused by the lower range of %ctDNA (0-44%, mean ~7.33%) compared to the VPC cohort (0–88.90%, mean ~38.46%). Still, high overall accuracy of 0.92 was achieved in these samples of lower genetic alteration load (Supplementary Fig. 5F, G). Similarly, misclassified regions have significantly lower CNA degree (Supplementary Fig. 5F) and the high proportion of correctly predicted neutral regions contributed to the overall high prediction accuracy (Supplementary Fig. 5G). Taken together, we showed that cfMeDIP-seq data can be used to reliably predict sample CNA.

**Table 1:** The performance and accuracy of CNA prediction in mCRPC samples from the VPC cohort

	Precision	Specificity	Sensitivity	F1
Gain	0.66	0.98	0.53	0.59
Neutral	0.88	0.49	0.96	0.92
Loss	0.82	0.99	0.44	0.58
Mean	0.79	0.82	0.65	0.70
Accuracy	0.86			

## Discussion

In this work, we provide a genome-wide methylation analysis using the cell-free DNA isolated from the plasma of patients with localized and metastatic PCa. While the cfDNA methylation profile has been extensively studied using targeted analysis for early-stage, non-metastatic PCa^10,49^, evaluation in the advanced, metastatic disease at the genome scale is still sparse^10,49^. The mCRPC samples were collected from three independent studies. Using a subset of the data, we built a predictor to distinguish localized from metastatic samples that can be generalized to the remaining dataset. We achieved consistent high prediction accuracy on the three independent cohorts, suggesting that the methylation profiles are indeed capturing common features shared among mCRPCs. Considering the highly heterogeneous nature of mCRPCs, such consistent and highly sensitive performance is of particular interest and suggests potential clinical utility, such as early detection of oligometastasis. Oligometastatic disease is increasingly recognized as an independent state with low-volume metastasis^50^. Here, we show the ability of the cfDNA methylome in detecting metastasis with very low to undetectable levels of genetic alterations, a characteristic likely shared with oligometastasis. Specialized effort is needed to explore and directly validate its utility in such cases.

We observe pervasive hypermethylation in metastasis compared to localized PCa. The preponderance of hypermethylation in mCRPC compared with localized samples may be disease-specific, as a proportional amount of hypo and hyper DMRs has been observed in pancreatic cancer, while preponderant hyper DMRs were observed in head and neck squamous carcinoma, with the same cfMeDIP-seq method^16,32^. These sites were enriched in gene promoters, CpG islands, and shores, sharing the same feature of frequent hypermethylation observed in the early stage of PCa^37,51^, suggesting progressive maintenance of dysregulated DNA methylation. Such noticeably higher number of hypermethylation sites seemingly contradict previous observations of global hypomethylation in metastatic PCa^37,51^. Several factors might contribute to these observed differences: (1) Most early studies assess the global methylation changes by analyzing the total methylated DNA and the results might not reflect changes at individual sites. (2) Different analytical methods might also result in paradoxical observations: while a meta-analysis showed that hypomethylation is associated with PCa^51^, a more recent pan-cancer analysis identified more hypermethylated sites^52^. (3) Complexity of the cfDNA origin in plasma can further contribute to the observed disparities. Compared to metastasis, localized cancers are known to have lower ctDNA fraction, thus comparisons of plasma samples are likely to simultaneously capture differences between normal and tumorous tissues. Despite the overall consistency observed between tissue and cell-free methylomes, such disparities highlighted the need to directly analyze the cell-free methylomes.

In contrast to hyper-DMR, we observed no correlation between the overall hypo-DMR methylation and %ctDNA, suggesting non-tumor source, such as TME, of such variations. Tumor grows in a specialized microenvironment and induces extensive remodeling, including changes in their DNA methylation profiles, in the surrounding non-cancerous cells. These changes in the TME may in turn alter the cell-free methylome^53^. Indeed, we identified sites that are correlated with ALP and DHL, indicators associated with diseases residing in different environments, potentially reflective of TME-derived changes. Future effort is needed to dissect the cell sources of tumor-associated cell-free methylome changes.

The enrichment of MHC-I immune gene signature in GR-DMR high patients and the inverse association with survival in localized and metastatic PCa are intriguing. While glucocorticoids are frequently prescribed as co-medication for the treatment of solid tumors including PCa, recent studies showed that high GR expression was associated with worse outcome in mCRPC^54,55^. It is worth noting that when investigating the association between GR-DMR methylation and %ctDNA, the signals seem to be derived from different distributions, suggesting the existence of multiple subtypes. Such mixture can also be the combined result of the tumor and non-tumor TME components, as tumor cells are able to alter the DNA methylation profile of the TME, which can further change the cfDNA methylome^56,57^. Dissecting the cells of origin of GR-DMR methylation can help shed light upon its underlying functional mechanisms. Moreover, we only observed moderate negative correlation in GR-DMR methylation and GR gene expression, whether the GR-DMR function through mediating GR transcription warrants further investigation.

Hypomethylated sites identified in this study are enriched in repeat and particularly pericentromeric regions. While global hypomethylation of repeat classes like LINE1 elements in cancer is commonly acknowledged, pericentromeric DNA sequences are less investigated. Pericentromeric DNA hypomethylation in other solid tumors like breast cancer and melanoma has been associated with chromosome instability^58,59^, in line with the increased genomic instability observed in metastatic PCa. Analysis of the DNA methylation in pericentromeric regions had been focused on sequences in chromosome 1, while we showed here such hypomethylation can encompass most chromosomes and is more prevalent than previously realized. Additional effort is needed to understand the functional implication of these epigenetic dysregulations.

In summary, we identified consistent methylation changes and created classifiers that can distinguish between localized and metastatic samples with high accuracy. The results presented multiple insights into the disease biology and demonstrated the biomarker potential for detecting metastatic lesions with a minimally invasive, highly sensitive, and cost-effective strategy.

## Methods

This work complies with all relevant ethical regulations. All patients provided informed written consent and all samples were obtained upon approval of the institutional ethics committee and Research Ethics Board at the University Health Network (UHN) and the University of British Columbia (UBC), with compliance with all relevant ethical regulations. CPC and Barrier samples were retrieved from the UHN GU Biobank (REB file numbers: 11-0024(CPC) and 13-7122(Barrier)). VPC cfDNA from plasma was retrieved from the Vancouver Prostate Centre (VPC), UBC (REB file number: H18-00944). The WCDT cfDNA samples were retrieved from the University of California San Francisco (UCSF). The OHS samples were retrieved from the Ontario Institute for Cancer Research (OICR).

### Contact for reagent and resource sharing

Further information and requests for resources should be directed to and will be fulfilled by Lead Contact, Housheng Hansen He (hansenhe@uhnresearch.ca).

### Experimental models and subject details

#### Method details

##### Cell-free DNA isolation from plasma

Peripheral blood was collected from cancer patients using EDTA anticoagulant tubes. Plasma samples were isolated from whole blood using the UHN Biobank centrifugation protocol and stored at the UHN Biobank. 500 µl–1 ml plasma aliquots retrieved from the Biobank were immediately stored at −80 °C for short-term use. The cfDNA was isolated from plasma using the QIAamp Circulating Nucleic Acid Kit (Qiagen) according to the manufacturer’s protocol and quantified by Qubit (Thermo Fisher Scientific) before use. Within each experimental batch, samples were randomized by disease status and performed blinded during cfMeDIP-seq wet-lab processing.

##### Cell-free methylated DNA immunoprecipitation and sequencing

To prepare cfMeDIP libraries for sequencing, the original cfMeDIP-seq protocol was used^16^ on 5 ng of input cfDNA per sample. First, the samples underwent library preparation using Kapa HyperPrep Kit (Kapa Biosystems) for end-repair and A-tailing, following the manufacturer’s instructions. Samples were then ligated to 0.181uM of NEBNext adaptor (NEBNext Multiplex Oligos for Illumina kit, New England Biolabs) by incubating at 20 °C for 20 mins. The DNA was then purified with AMPure XP beads (Beckman Coulter). The library was then digested using USER enzyme (New England Biolabs) and then purified with Qiagen MinElute PCR purification kit (MinElute columns).

The prepared libraries were then combined with 95 ng of filler DNA (λ phage), and then MeDIP was performed using the Diagenode MagMeDIP kit (C02010021) using a previously published protocol^26^. The filler DNA consists of a mixture of unmethylated and in vitro methylated λ amplicons of different CpG densities: 1 CpG site, 5 CpG sites, 10 CpG sites, 15 CpG sites, and 20 CpG sites; all similar in size to cfDNA. This filler DNA ensures a constant ratio of antibody to input DNA and minimizes non-specific binding by the antibody and prevents cfDNA loss due to binding to plasticware. Once the prepared library and filler DNA were combined, 0.3 ng of control methylated and 0.3 ng of control unmethylated *Arabidopsis thaliana* DNA and the buffers from the MagMeDIP kit were added, as per the manufacturer’s instructions. The mixture was heated to 95 °C for 10 min, then immediately placed on ice for 10 mins. Each sample was partitioned into two 0.2 ml PCR tubes: one for 10% input control (7.9 µl) and the other for the sample to be subjected to immunoprecipitation (79 µl). The included 5mC monoclonal antibody (C15200081) from the MagMeDIP kit was diluted to 1:15 before adding it to the immunoprecipitation sample. MagMeDIP magnetic beads were then washed 2× with prepared buffers from the kit and added to the sample before incubation at 4 °C for 17 h with rotation. The samples were purified using the Diagenode iPure Kit v2 (C03010015) and eluted in 50 µl of buffer C.

Quality control 1 (QC1) was performed by qPCR to detect recovery of the spiked-in methylated and unmethylated *A. thaliana* DNA. The recovery of methylated *A. thaliana* DNA should be >20%, unmethylated *A. thaliana* DNA should be <1% (relative to the input control and adjusted to input control being 10% of the overall sample), and the specificity of the reaction should be >99% (1−[recovery of spike-in unmethylated DNA/ recovery of spike-in methylated DNA] × 100) to proceed. The PCR cycle number for library amplification was determined by qPCR (QC2) and should be <15 cycles to proceed, and the samples were amplified using Kapa HiFi Hotstart Mastermix and NEBNext multiplex oligos, added to a final concentration of 0.3uM. The final libraries were amplified as follows: activation at 95 °C for 3 min, # cycles: 98 °C for 20 s, 65 °C for 15 s, and 72 °C for 30 s, and a final extension of 72 °C for 1 min. The amplified libraries were purified using MinElute columns, then size selected to remove adaptor dimers by either using 3% Nusieve GTG agarose gel and subsequent get cutting, or Pippin Prep (Sage Science) following the manufacturer’s instructions. All the final libraries were then checked at TapeStation (Agilent) for library concentration, correct sizing, then pooled with six other cfMeDIP samples with different NEBNext barcodes. The pool of seven samples (per lane) was sequenced at 150 bp paired-end on Illumina HiSeq X ten.

##### Publicly available data

The TCGA prostate adenocarcinoma (PRAD) 450 K methylation data (hg19 based) were downloaded from the TCGA Data Portal (https://tcga-data.nci.nih.gov/tcga/), including 50 normal tissue and 489 primary tumor samples. Associated clinical data and normalized gene expression were also obtained. The CPC-GENE (Canadian Prostate Cancer Genome Network) 450 K methylation data from 286 patients with localized prostate adenocarcinoma, matching normalized gene expression and clinic information (hg19 based) were obtained from the previous publication^22^. Processed whole-genome bisulfite sequencing (WGBS, hg38-based), RNA-seq, and clinical data for 194 Asian patients with localized tumors and matched healthy tissue were obtained from CPGEA^46^, the Chinese Prostate Cancer Genome and Epigenome Atlas (http://www.cpgea.com). Processed WGBS and matched RNA-seq data (hg38-based) for 100 WCDT mCRPC (West Coast Dream Team, metastatic castration-resistant PCa) were obtained from the previous publication^21^. The WGBS data from WCDT and CPGEA cohorts were converted to methylation values for the same 300 bins used in cfMeDIP-seq data analysis. Methylation value was defined as the total number of methylated counts divided by the total number of (methylated and unmethylated) counts in this 300 bp region. The hg38 genome coordinates were converted to hg19 using liftOver (v1.10.0) R package with a chain file retrieved from the USCS genome browser (https://genome.ucsc.edu/).

Genes (CTAG1B, TSPY1, MAGEA3, and PAGE1) with promoter hypomethylation in PCa were obtained from the previous publication^37^. A total of 136 PCa driver genes were obtained from previous publications and DriverDBv3, consisting of 57 oncogenes and 79 tumor suppressor genes^38,39^. Bins within the promoter region (1 kb upstream of TSS) of these genes were compared in the cfMeDIP-seq data.

##### Quantification and statistical analysis

Statistical analyzes were performed using R statistical environment (v3.6.1) (R Core Team, 2019). All tests were two-sided unless otherwise specified. The type of test method used for statistical analysis was specified in the text where the results were described and details for the test were explained in the relevant figure legend and method section.

##### Sequencing data preprocessing

Human genome (hg19/ GRCh37) was downloaded from the University of California Santa Cruz (UCSC) genome browser (https://genome.ucsc.edu/). The quality of raw reads was assessed using FastQC^60^ (v0.11.5) and MultiQC^61^ (v0.8). Trim Galore (v0.5.0, https://github.com/FelixKrueger/TrimGalore) (“--phred33 --stringency 3 --length 20 -e 0.1”) was used to remove adapters and trim poor-quality sequencing reads. After trimming, the reads were aligned to the human reference genome using BWA^62^ (v0.7.15) with default parameters. SAMtools^63^ (v1.3.1) with default settings was used to convert Sam to Bam format, filter out duplicates, sort and index the files and provide mapping statistics for the output. For paired-end data, we filtered for properly paired alignments using SAMtools^63^ (“-h -f 2 -F 512”).

##### Fragment size analysis

The fragment sizes for each sample were calculated using the CollecInsertSizeMetrics function from Picard (v2.6.0) (https://github.com/broadinstitute/picard) on the sorted bam files, setting the minimum percentage option to 0.5. The longer fragment ratio was defined as the proportion of the number of reads from 170 bp to 210 bp to the number of reads from100bp to 210 bp.

For fragmentation profiles, customized scripts from previous study^36^ (http:github.com/Cancer-Genomics/delfi_scripts) were applied on sorted bam files to calculate fragment ratios in 5-Mb bins.

##### Calculation of DMRs

DMRs between metastatic (67 VPC cohort samples) and localized (30 CPC cohort samples) PCa were identified using DESeq2^64^ (v1.24.0) while controlling for age differences. Before detecting DMRs, the count generated by MEDIPS^65^ (v1.34.0) was first converted into reads per kilobase per million mapped reads (RPKM) using the total number of reads as the library size. Only bins with higher than 5 RPKM in at least one sample across all PCa samples were retained (non-low coverage bins). The raw counts of these non-low coverage bins were used as input for DESeq2^64^. Bins with Benjamini-Hochberg adjusted *p*-value <0.05 and absolute fold change greater than 2 were nominated as DMRs.

##### DMRs annotation and enrichment analysis

The genomic annotations of DMRs were obtained using the R packages annotatr^66^ (v1.12.1), TxDb.Hsapiens.UCSC.hg19.knownGene (v3.2.2) and org.Hs.eg.db (v3.10.0) from Bioconductor^67,68^. ChIP-seq data for important TFs were collected from the gene expression omnibus (GEO) (Supplementary Data 4). Customized annotation using the TF ChIP-seq data was performed using GenomicRanges^69^ (v1.38.0). To assess whether DMRs are enriched or depleted in the annotated regions, adjacent regions of DMRs were first merged using the ‘reduce’ function of GenomicRanges^69^. Association analysis was then performed by regioneR^70^ (v1.16.2) with a permutation test (1000 iterations). The 33,740 300 bp non-low bins were used as background regions. *P* value of 0.05 was used as a cutoff for significance.

##### Differential gene expression analysis between high and low GR-DMR methylation groups

The most noticeable hyper-DMR site (chr5:142782301-142782600, referred to as “GR-DMR”) in metastasis compared to localized plasma samples is in the promoter of GR (also known as *NR3C1*) gene. To investigate the transcriptional effect of this site, we used public datasets from the TCGA, CPGEA, CPC, and WCDT cohorts^20,21,46,47^. Considering that this site has low coverage in most of the samples from these cohorts, the top 10 samples with the highest and lowest methylation values were selected for comparison. Samples were grouped into GR-high and GR-low groups according to methylation levels on the GR site, and differentially expressed genes (DEGs) were determined using matched RNA-seq data. The DEGs were identified using DESeq2 (v1.24.0)^64^ with --FDR = 0.05, --log2FC = 1”.

For the 450 K DNA methylation array from TCGA PRAD and CPC cohorts^22^, the beta value of the CpG site overlapping with this DMR region was regarded as the methylation signal of this region. The schematics of the GR gene were plotted using the R package Gviz (v1.30.3).

##### Gene enrichment analysis

Gene enrichment analysis was performed using TCGAbiolinks^71^ (v2.14.0) with an FDR of 0.01 as cutoff. For DEGs from tissue RNA-seq data, the upregulated and downregulated genes were analyzed separately in the functions or pathways enrichment analysis. For DMRs from plasma cfMeDIP-seq data, genes with DMRs located within the 5Kb upstream of transcription start sites were used.

##### Predicting CNAs using plasma cfMeDIP data

To assess the ability of cfMeDIP-seq data on determining CNA events of the data, the CaSpER^48^ (v0.1.0) R package was used for analysis. Briefly, CaSpER first preforms data smooth on three different length scale. CNA detection was then performed by taking into account both the three-scale smoothed DNA methylation signals and whole-genome allelic shift profiles inferred from plasma cfMeDIP-seq data. On each scale: 1 CNA states (gain, loss, or neutral) were assigned using Hidden Markov Model (HMM). 2 The genome-wide allelic shift profiles were estimated using Gaussian mixture model to correct CNA predictions with relatively low evidence of methylation signals. In our analysis, localized samples from the CPC cohort were used as reference. The final consistent CNA calls were defined as CNA identified by at least six times from all pairwise scale comparisons. For the VPC cohort, inferred CNA states were compared to the gold standard calls from panel sequencing obtained previously^11^.

##### Machine learning for diagnostic classification

To evaluate the performance for diagnostic tumor classification based on plasma cfMeDIP-seq, we randomly selected an equal number of unique samples from the localized and mCRPC cohorts as training sets and use the remaining unique patients different from training sets as testing sets. For mCRPC samples from the Barrier and WCDT cohorts, 3 out of 14 and 19 out of 22 unique samples were used, respectively. Feature selection and model construction were performed in training sets, and the model performance was evaluated in testing sets. Briefly, to reduce the impact of technical factors, first, the methylated values were corrected and normalized using sva^72^ (v3.32.1) and DESeq2^64^ (v1.24.0), considering sequencing batch as a confounder. Second, DMRs between localized and metastatic patients from the training set were identified using DESeq2^64^ (v1.24.0) as described above. Third, the top 150 hyper-DMRs and hypo-DMRs were then selected by measuring information gain and used to build a classification model using randomForest (v4.6.14) (https://www.stat.berkeley.edu/~breiman/RandomForests/). Finally, the performance of the randomForest classifier was evaluated on the testing set. The AUROC (area under the receiver operating characteristics) curves were estimated using the probability from the random forest model and used for visualization. This procedure was repeated 50 times.

##### Performance assessment

We used several evaluation metrics to assess the classification performance of localized and metastatic PCa, including sensitivity (1), specificity (2), precision (3), accuracy (4), and F1 score (5). These indicators were also employed in the evaluation of CNA prediction by cfMeDIP-seq profiles.

1 $${{{{{\rm{Sensitivity}}}}}}={{{{{\rm{TP}}}}}}/({{{{{\rm{TP}}}}}}+{{{{{\rm{FN}}}}}})$$

2 $${{{{{\rm{Specificity}}}}}}={{{{{\rm{TN}}}}}}/({{{{{\rm{TN}}}}}}+{{{{{\rm{FP}}}}}})$$

3 $${{{{{\rm{Precision}}}}}}={{{{{\rm{TP}}}}}}/({{{{{\rm{TP}}}}}}+{{{{{\rm{FP}}}}}})$$

4 $${{{{{\rm{Accuracy}}}}}}=({{{{{\rm{TP}}}}}}+{{{{{\rm{TN}}}}}})/({{{{{\rm{TP}}}}}}+{{{{{\rm{TN}}}}}}+{{{{{\rm{FP}}}}}}+{{{{{\rm{FN}}}}}})$$

5 $${{{{{\rm{F1}}}}}}\,{{{{{\rm{score}}}}}}=2{{{{{\rm{TP}}}}}}/(2{{{{{\rm{TP}}}}}}+{{{{{\rm{FP}}}}}}+{{{{{\rm{FN}}}}}})$$

TP stands for true positive, TN for true negative, FP for false positive, and FN for false negative. Sensitivity (also known as Recall) indicates the fraction of positive patients that are correctly predicted. Specificity (also known as Selectivity) indicates the fraction of negative patients that are correctly predicted. Precision indicates the fraction of correctly identified positive patients to the total identified positive patients. Accuracy indicated the fraction of correctly identified patients to the total observed patients. F1 score is a comprehensive indicator calculated by combining precision and sensitivity, with a higher score representing better performance. AUROC curve was calculated using the R package ROCR (v1.07)^73^.

##### Survival analysis

Kaplan–Meier plots were created using survival (v3.1.8) and survminer (v0.4.6), in which *p* value of survival between two groups was calculated using a log-rank test (cutoff *p* value = 0.05). For overall DMR analysis, we first calculated a hyper:hypo DMR ratio by dividing the mean methylated values of all hyper-DMRs by the mean methylated value of all hypo-DMRs. mCRPC samples from the VPC cohort were then split into high and low according to the median ratio (0.9786). For the fragment analysis, the fragment value was defined as the fragment ratio of the number of reads from 170 bp to 210 bp to the number reads from 100 bp to 210 bp. Patients were classified into shorter or longer fragment groups based on the median value of the fragment ratio. For GR-DMR-related analysis, we divided the patients into high GR-methylated and low GR-methylated groups according to the median value of the GR-DMR methylation. A similar analysis was performed for mRNA expression between patients with high and low GR gene expression.

The overall survival (OS) and PFS survival were used for the metastatic samples from the VPC cohort, and the OS also was used for the metastatic samples from the WCDT cohort. The biochemical recurrence-free survival was used for the localized samples from CPC and CPGEA cohorts. The clinic endpoints of recurrence-free survival were used for the 498 TCGA samples.

##### Repeat region analysis

To analyze repeat regions, we used a peak strategy to summarize cfMeDIP-seq data signal. We first filtered bam files using samtools (v1.3.1) to obtain high-quality primary alignments (-F 1804). Next, we used the MACS^74^ (v2.2.5) “callpeak” function to generate narrowPeak on all samples with --SPMR parameter to generate a normalized pileup file. Pileup files from the peak calling step were converted to bigWig files using the ucsctools (v378). Peak files from all samples were merged to create a peak catalog. Mean signal intensities were summarized for each of the intervals in the peak catalog using bwtool (v1.0) from sample bigWig files.

### Reporting summary

Further information on research design is available in the Nature Research Reporting Summary linked to this article.

## Supplementary information

Supplementary Information

Description of Additional Supplementary Files

Supplementary Data 1

Supplementary Data 2

Supplementary Data 3

Supplementary Data 4

Reporting Summary

## Data Availability

The raw cfMeDIP-seq data generated in this study have been deposited in the European Genome-Phenome Archive (EGA) database under the study accession code EGAS00001005522 and the dataset accession codes EGAD00001007972, EGAD00001008711, EGAD00001008712, EGAD00001008713, EGAD00001008737. The raw data are available under restricted access due to them containing identifying information that could compromise patient privacy. Access can be obtained by contacting the data access committee listed on the EGA page and according to the EGA guideline. There are no restrictions on data access application. Applications will be reviewed monthly, and once all patient privacy and data transfer documents are completed; we will notify EGA within two-weeks to allow data downloading. Immediately upon receipt of our notification, EGA will create an account for the applicant to download data and the timeframe to download data will be in accordance with EGA guidance. The processed bin level raw count data are provided in Supplementary Data 2 on Open Science Framework (OFS, https://osf.io/97tqk/); normalized peak level intensity is available in Supplementary Data 3 on OFS (https://osf.io/97tqk/). Source data are provided with this paper. The human hg19 reference genome and the chain file for liftOver was downloaded from the UCSC genome browser (https://hgdownload.soe.ucsc.edu/goldenPath/hg19/bigZips/genes/ and https://hgdownload.soe.ucsc.edu/goldenPath/hg38/liftOver/). The public panel gene analysis data for the VPC cohort used in this study are available in the EGA database under accession code EGAS00001003113^23^. The publicly available data from the CPC cohort used in this study are available in the EGA database under accession code EGAS00001000900^20,22,24^. The publicly available data from the CPGEA cohort used in this study are available in the National Genomics Data Center (NGDC) under the accession code PRJCA001124, and the processed data can be accessed at: http://www.cpgea.com^46^. The publicly available data from the WCDT cohort used in this study are available in the database of Genotypes and Phenotypes (dbGAP) under the accession code phs001648, and the processed data can be accessed at: http://davidquigley.com/prostate.html^21^. The publicly available data TCGA data used in this study are available in the Broad Institute FireBrowse portal (http://firebrowse.org/?cohort=PRAD)^47^. Three genes with promoter hypomethylation in prostate cancer were obtained from a previous report^37^. A list of 136 prostate cancer driver genes was obtained from previous publication^38^ and database DriverDBv3^39^ (http://driverdb.tms.cmu.edu.tw/api/get_source_file?type=txt&cate=Cancer&symbol=250005866&tab=summary&file=summary_tab.txt). The remaining data are available within the Article, Supplementary Information, or Source Data file. Source data are provided with this paper.

## Source data

Source Data

## Code availability

All R packages used are available online as described in the method section. Customized code for data processing and visualization can be accessed on github: https://github.com/nikou123456/cfMeDIP_PCa.
