# Supplementary material for: The cell-free DNA methylome captures distinctions between localized and metastatic prostate tumors
Source: Nat Commun. 2022 Oct 29;13:6467. doi: 10.1038/s41467-022-34012-2 (PMC9617856; doi:10.1038/s41467-022-34012-2)
Supplement: Supplementary file 1 — Supplementary Information [file 41467_2022_34012_MOESM1_ESM.pdf]

Supplementary information for

The cell free DNA methylome captures distinctions between localized and metastatic prostate tumors

Sujun Chen[1,2,3,*], Jessica Petricca[1,2,*], Wenbin Ye[1,4,5,*], Jiansheng Guan[1,6], Yong Zeng[1], Nicholas Cheng[2], Linsey Gong[1,2], Shu Yi Shen[1], Junjie T. Hua[7,8], Megan Crumbaker[9], Michael Fraser[1], Stanley Liu[2,10,11], Scott V. Bratman[1,2], Theo van der Kwast[2,12], Trevor Pugh[1,2], Anthony M. Joshua[12], Daniel D. De Carvalho[1,2], Kim N. Chi[13], Philip Awadalla[14], Guoli Ji[4, 5, #], Felix Feng[7,8,15,16,#], Alexander W. Wyatt[17,#], Housheng Hansen He [1,2,#]

Supplementary Figures

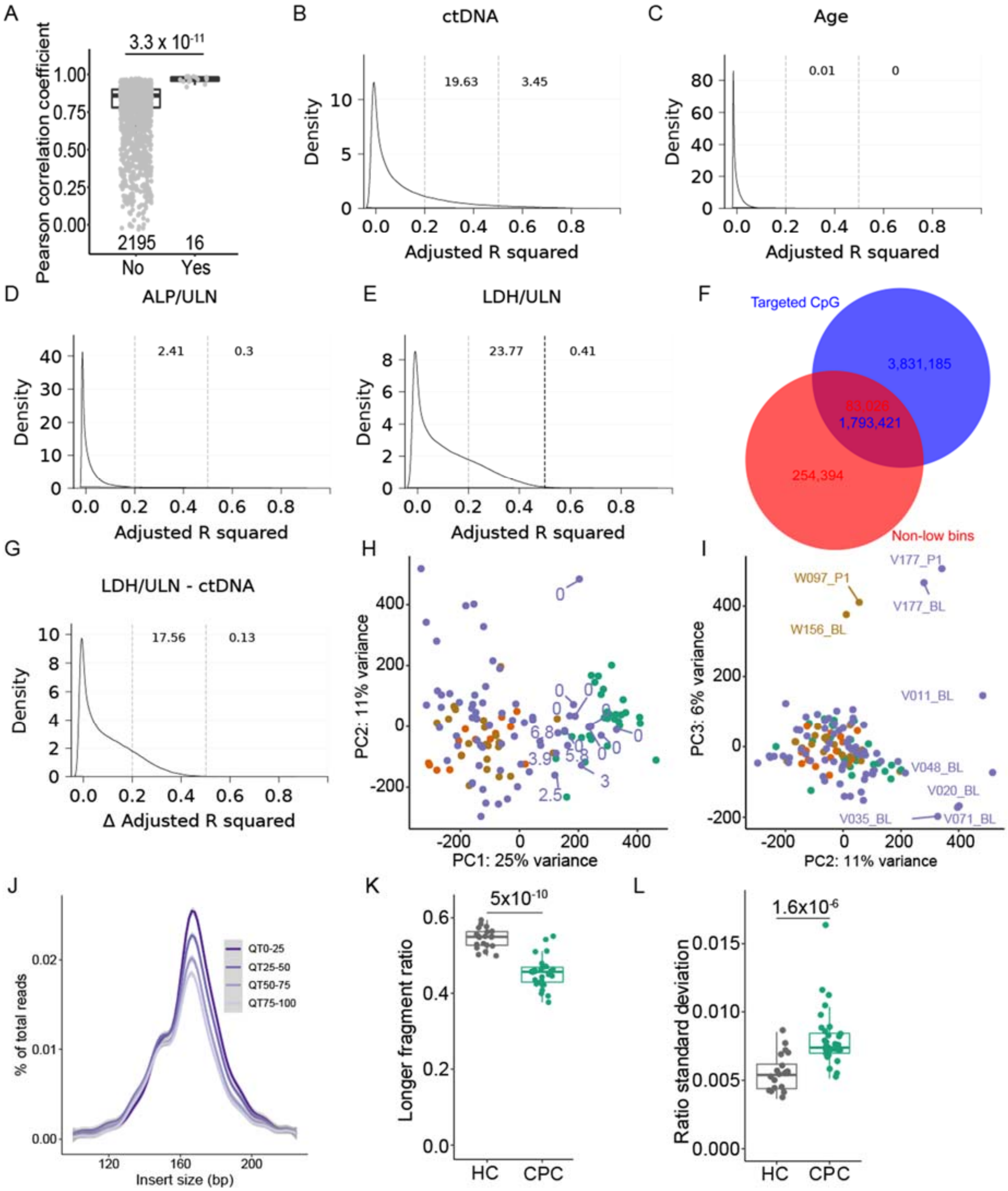

Supplementary Figure 1. cfMeDIP data captures variation beyond the tumor ctDNA. Related to Figure 1.

A) Pairwise sample correlation using cfMeDIP data, matched samples collected from the same patient at different timepoints were compared with the others. P value = 3.3×10^{-11} (two-sided Mann-Whitney U test). Box plots represent median values and 0.25 and 0.75 quantiles. Whiskers represent 1.5× interquartile range (IQR). X = 2195 and 16 independent observations for the “No (matched)” and “Yes (unmatched)” groups, respectively. Density distribution of the adjusted R squared from linear model fitted using %ctDNA B), age C), ALP/ULN D) and LDH/ULN E) as a variable for all the non-low bins. Numbers above the plot show the percentage of bins with > 0.2 and > 0.5 adjusted R squared values. ALP and LDH levels are measured as a ratio to the upper limit of normal (ULN) level. F) Overlap between the non-low bins and the targeted CpG sites in the Roche Epi CpGiant Probes. G) Density distribution of the delta adjusted R squared between a multi-variate linear model using both %ctDNA and LDH/ULN as variables and a univariate linear model with %ctDNA. Numbers above the plot show the percentage of bins with > 0.2 and > 0.5 delta adjusted R squared values. Sample distribution between PC2 and PC1 H); PC2 and PC3 I). J) Size distribution of estimated fragment length for mCRPC samples from the VPC cohort, samples grouped into four quartile groups according to %ctDNA levels, with QT0-25 corresponds to samples with lowest %ctDNA. Distribution of the longer cfDNA fragments K) and standard deviation of fragment ratio L) within a sample. Two-sided Mann-Whitney U test was used to calculate pairwise p-values between localized samples from the CPC and the healthy control (HC) samples from Burgener et al[1]. Box plots represent median values and 0.25 and 0.75 quantiles. Whiskers represent 1.5× interquartile range (IQR). X = 20 and 30 independent experiments for the HC and CPC cohorts, respectively, in K) and L). Source data for Supplementary Figures 1A, Supplementary Figures 1H-I and Supplementary Figures 1K-L are provided as a Source Data file.

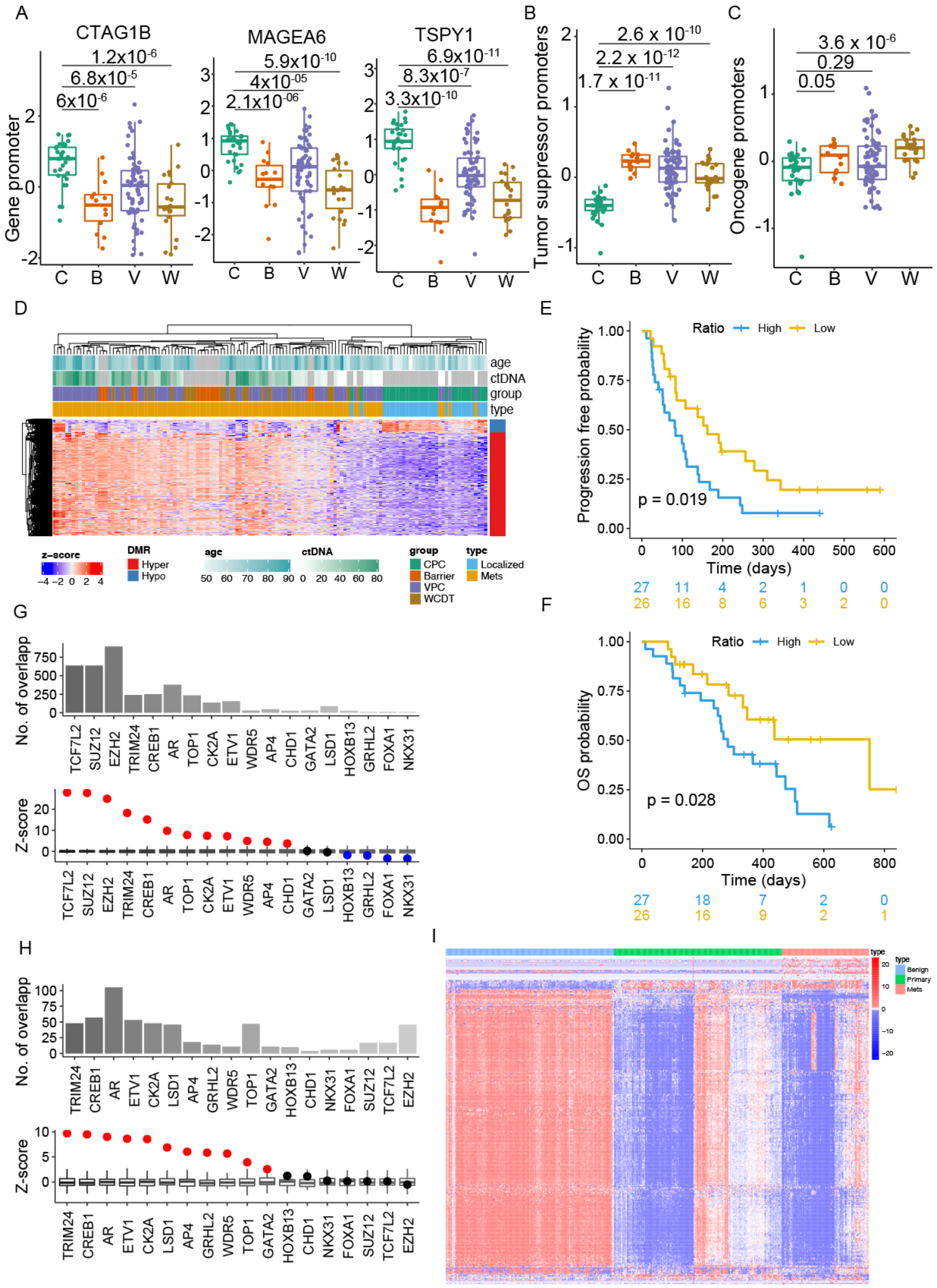
A
CTAG1B
1.2x10-6
6.8x10-5
6x10-6
MAGEA6
5.9x10-10
4x10-05
2.1x10-06
TSPY1
6.9x10-11
8.3x10-7
3.3x10-10
Gene promoter
C B V W
B
2.6 x 10-10
2.2 x 10-12
1.7 x 10-11
Tumor suppressor promoters
C
3.6 x 10-6
0.29
0.05
Oncogene promoters
D
age
ctDNA
group
type
z-score
DMR
Hyper
Hypo
group
CPC
Barrier
VPC
WCDT
type
Localized
Mets
E
Ratio High Low
Progression free probability
p = 0.019
Time (days)
F
Ratio High Low
OS probability
p = 0.028
Time (days)
G
No. of overlapp
Z-score
TCF7L2 SUZ12 EZH2 TRIM24 CREB1 AR TOP1 CK2A ETV1 WDR5 AP4 CHD1 GATA2 LSD1 HOXB13 GRHL2 FOXA1 NKX31
H
No. of overlapp
Z-score
TRIM24 CREB1 AR ETV1 CK2A LSD1 AP4 GRHL2 WDR5 TOP1 GATA2 HOXB13 CHD1 NKX31 FOXA1 SUZ12 TCF7L2 EZH2
I
type
Benign
Primary
Mets

Supplementary Figure 2. Characterization of DMRs between localized and metastatic samples. Related to Figure 2.

A) Average methylation level for bins located in the promoters of genes that were previously identified to be hypomethylated in metastatic tumors[2]. Average methylation level for bins located in the promoters of prostate cancer related tumor suppressors B) and oncogenes[3,4] C). Box plots represent median values and 0.25 and 0.75 quantiles. Two-sided Mann-Whitney U test was used to calculate pairwise p-values in A-C). Whiskers represent 1.5× interquartile range (IQR). X = 30, 14, 67 and 22 independent experiments for the CPC, Barrier, VPC and WCDT cohorts, respectively. D) Normalized methylation signal for DMRs across all four cohorts. Association of hyper-hypo DMR ratio with overall survival E) and progression free survival F) in mCRPC samples from the VPC cohort. Logrank test was used to calculate p-values. X = 27 and 26 independent observations for the high and low risk groups, respectively for both E) and F). Enrichment of important transcription factors (TF) on hyper- G) and hypo- H) DMRs. I) Differentially methylated peaks located within the 1Mb regions flanking the centromere in tissue WGBS data[5,6]. Source data for Supplementary Figures 2A-C are provided as a Source Data file.

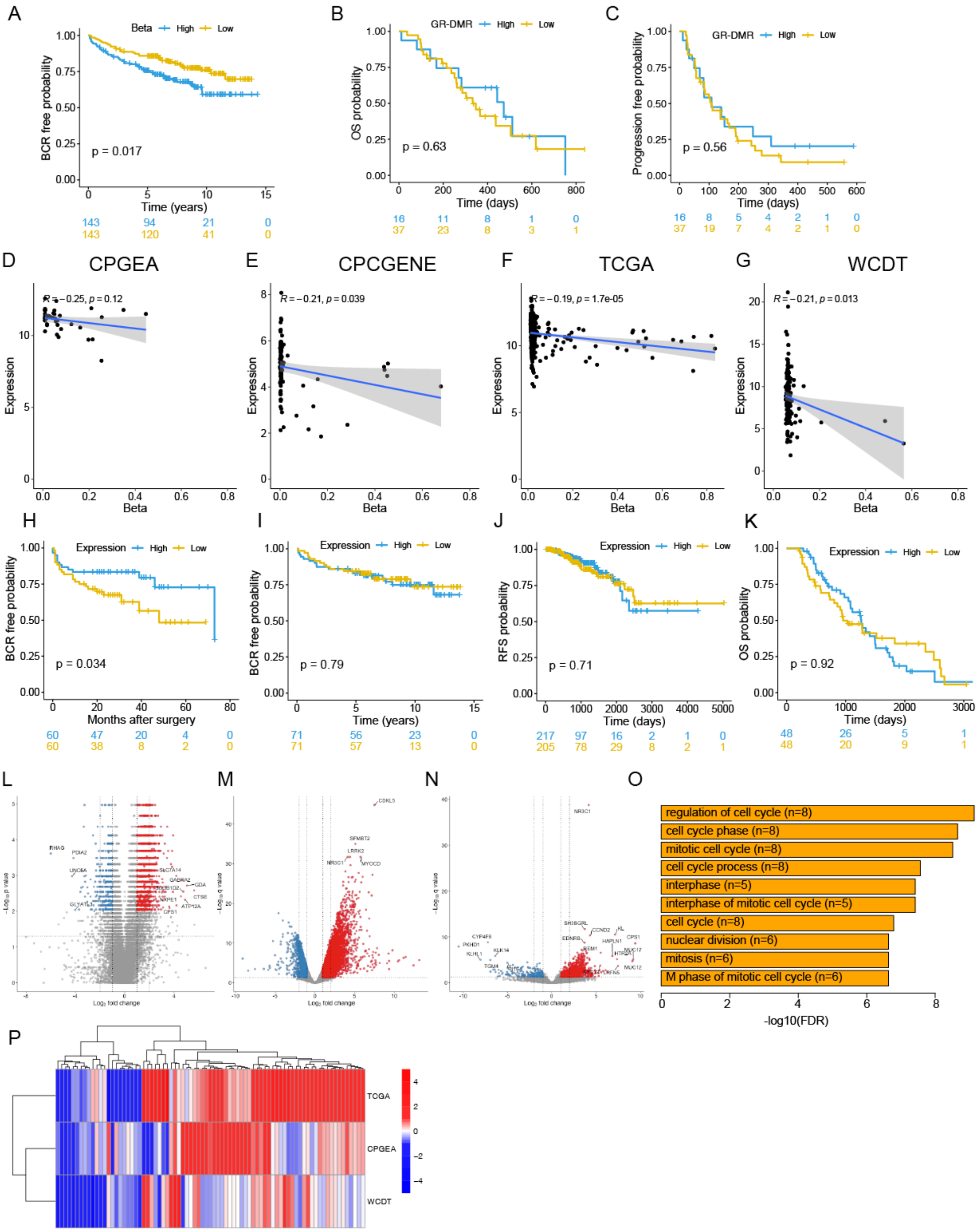

Supplementary Figure 3. Characterization of the GR site. Related to Figure 3

A) Association between GR site methylation and disease outcome in the localized samples from CPC cohort. BCR, biochemical recurrence. Association of GR site methylation measured by cfMeDIP-seq data with overall survival B) and progression free survival C) in mCRPC samples from the VPC cohort. Logrank test was used to calculate p-values. X = 143 and 144; 16 and 37; 16 and 37 independent observations for the high and low risk groups in A), B) and C) respectively. Pearson correlation between GR expression and methylation in the localized samples from CPGEA D), CPC E), TCGA F) and mCRPC samples from WCDT G) cohorts. P-values were calculated using two-sided *t*-test. Association between GR gene expression and disease outcome in the localized samples from CPGEA H), CPC I), TCGA J) and mCRPC samples from WCDT K) cohorts. Logrank test was used to calculate p-values. X = 60 and 60; 71 and 71; 217 and 205; 48 and 48 26 independent observations for the high and low risk groups in H), I), J), K) respectively. Volcano plots showing the differential gene expression analysis in GR-DMR methylation high compared to low groups in CPGEA L), TCGA M) and WCDT N) cohorts. O) Gene ontology (GO) analysis showing the enrichment of Biological Process (BP) for genes up regulated in high GR-DMR methylation groups in CPGEA. P) Log2 transformed fold change in L-N) for genes in BP terms shown in O). Only genes that are differentially regulated in at least one of the comparisons are shown. Source data for Supplementary Figures 3D-G are provided as a Source Data file.

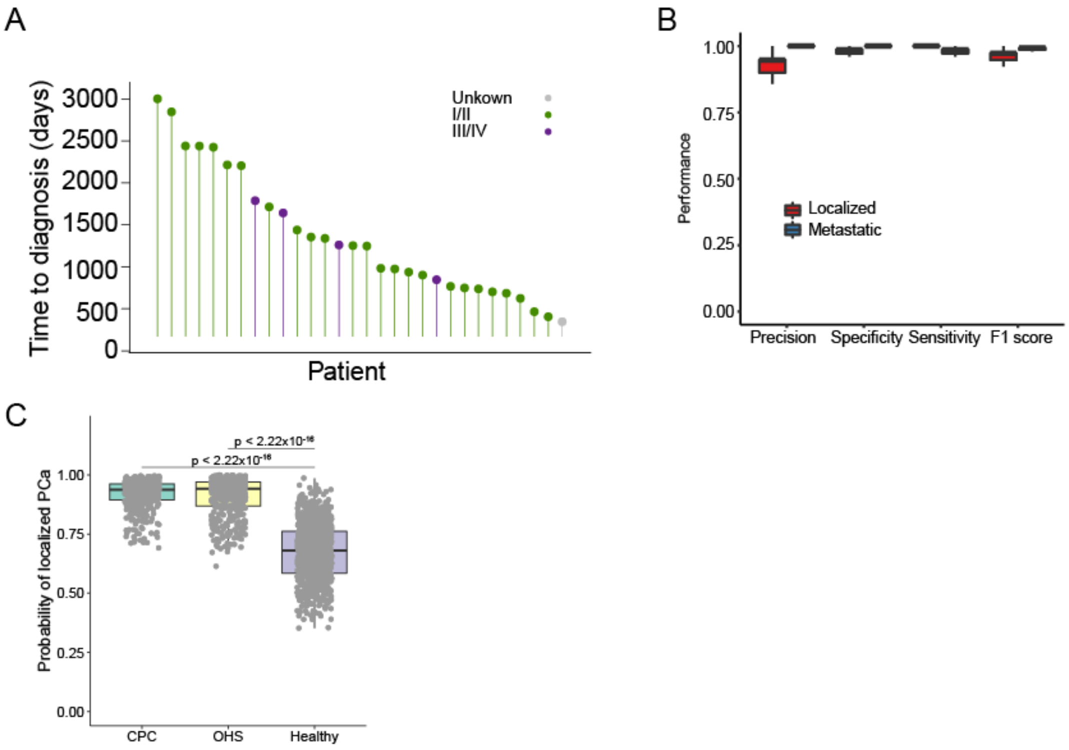

Supplementary Figure 4. Methylation profiles show distinction between samples of different disease status. Related to Figure 4

A) Time to diagnosis for samples from the Ontario Health Study (OHS). B) Performance statistics for the classifier distinguishing localized and metastatic samples for the 50 models. Box plots represent median values and 0.25 and 0.75 quantiles. Whiskers represent 1.5× interquartile range (IQR). X = 50 observations from 50 repeats for both “Localized” and “Metastatic” predictions. C) Probability distribution for localized samples from the CPC cohort and healthy controls from Burgener et al[1]. Box plots represent median values and 0.25 and 0.75 quantiles. Two-sided Mann-Whitney U test was used to calculate pairwise p-values. Whiskers represent 1.5× interquartile range (IQR). X = 450, 450 and 1,000 observations pooled from 50 repeats for the CPC, OHS and HC cohorts, respectively. Source data for Supplementary Figures 4B-C are provided as a Source Data file.

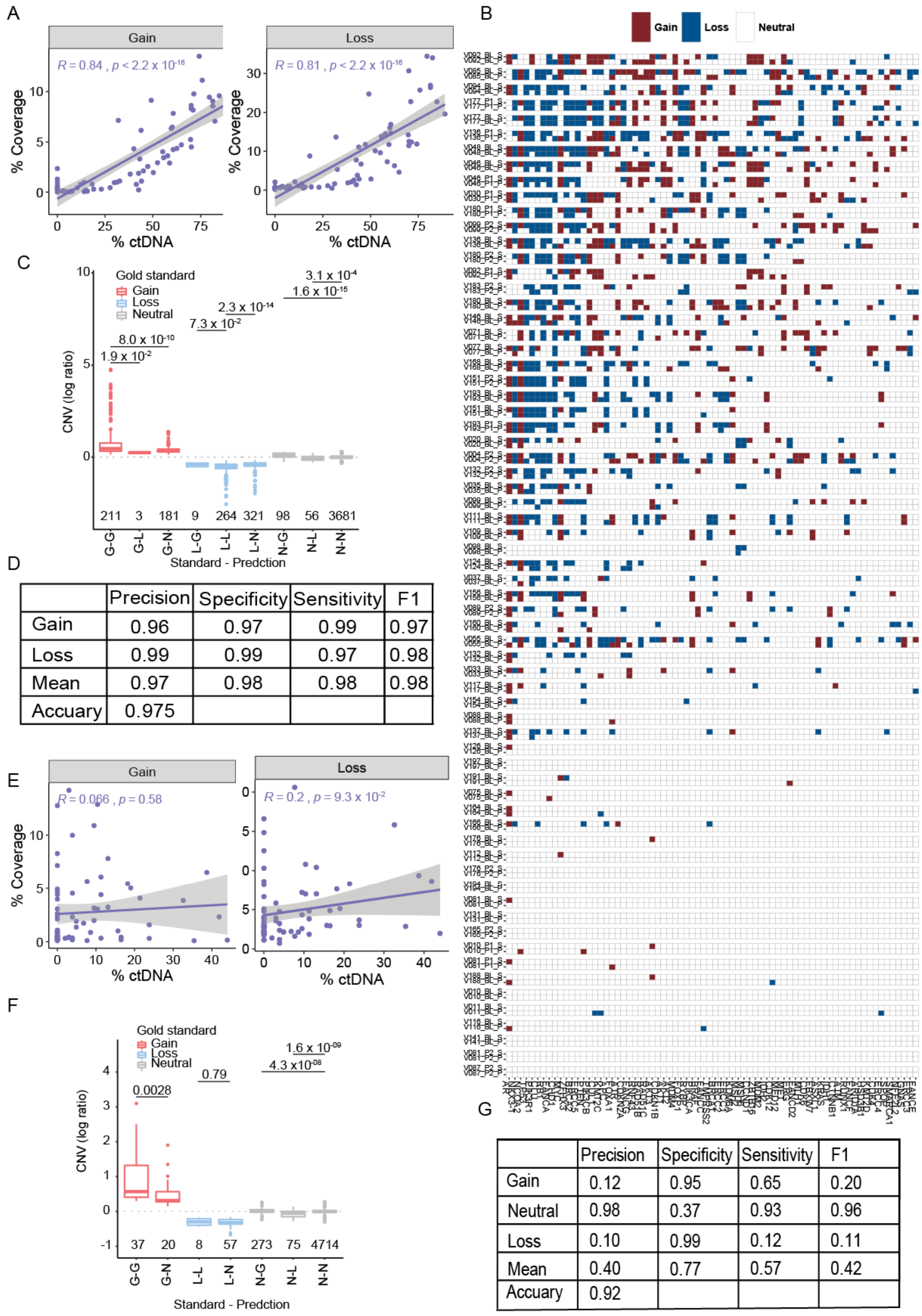

D

	Precision	Specificity	Sensitivity	F1
Gain	0.96	0.97	0.99	0.97
Loss	0.99	0.99	0.97	0.98
Mean	0.97	0.98	0.98	0.98
Accuary	0.975			

G

	Precision	Specificity	Sensitivity	F1
Gain	0.12	0.95	0.65	0.20
Neutral	0.98	0.37	0.93	0.96
Loss	0.10	0.99	0.12	0.11
Mean	0.40	0.77	0.57	0.42
Accuary	0.92			

Supplementary Figure 5. cfMeDIP-seq data can predict sample CNV with high accuracy. Related to Figure 5

A) Pearson correlation between CNA coverage and %ctDNA in mCRPC samples from the VPC cohort. P-value was calculated using two-sided *t*-test. B) Comparison of gene CNA between standard status obtained from previous panel sequencing and predicted results from cfMeDIP-seq data for the remaining 57 samples from Figure 5C. C) Distribution of CNA degree among different prediction types. Two-sided Mann-Whitney U test was used to calculate pairwise p-values between different types. Box plots represent median values and 0.25 and 0.75 quantiles. Whiskers represent 1.5× interquartile range (IQR). Number below each box represents the number of independent observations for each group. D) The performance and accuracy of CNA prediction for genes with altered copy number in mCRPC samples from the VPC cohort. E) Pearson correlation between CNA coverage and %ctDNA in mCRPC samples from the VPC-V cohort. P-value was calculated using two-sided *t*-test. F) Distribution of CNA degree among different prediction types for mCRPC samples from VPC-V cohort. Two-sided Mann-Whitney U test was used to calculate pairwise p-values. Box plots represent median values and 0.25 and 0.75 quantiles. Whiskers represent 1.5× interquartile range (IQR). Number below each box represents the number of independent observations for each group. G) The performance and accuracy for CNA prediction in mCRPC samples from the VPC-V cohort. Source data for Supplementary Figures 5A, Supplementary Figures 5C-F are provided as a Source Data file.

References:

1 Burgener, J. M. *et al.* Tumor-Naive Multimodal Profiling of Circulating Tumor DNA in Head and Neck Squamous Cell Carcinoma. *Clin Cancer Res* **27**, 4230-4244, doi:10.1158/1078-0432.CCR-21-0110 (2021).

2 Yegnasubramanian, S. *et al.* DNA hypomethylation arises later in prostate cancer progression than CpG island hypermethylation and contributes to metastatic tumor heterogeneity. *Cancer Res* **68**, 8954-8967, doi:10.1158/0008-5472.CAN-07-6088 (2008).

3 Du, M. *et al.* Plasma cell-free DNA-based predictors of response to abiraterone acetate/prednisone and prognostic factors in metastatic castration-resistant prostate cancer. *Prostate Cancer Prostatic Dis* **23**, 705-713, doi:10.1038/s41391-020-0224-4 (2020).

4 Liu, S. H. *et al.* DriverDBv3: a multi-omics database for cancer driver gene research. *Nucleic Acids Res* **48**, D863-D870, doi:10.1093/nar/gkz964 (2020).

5 Li, J. *et al.* A genomic and epigenomic atlas of prostate cancer in Asian populations. *Nature* **580**, 93-99, doi:10.1038/s41586-020-2135-x (2020).

6 Zhao, S. G. *et al.* The DNA methylation landscape of advanced prostate cancer. *Nat Genet* **52**, 778-789, doi:10.1038/s41588-020-0648-8 (2020).
