# Supplementary material for: The cell-free DNA methylome captures distinctions between localized and metastatic prostate tumors
Source: Nat Commun. 2022 Oct 29;13:6467. doi: 10.1038/s41467-022-34012-2 (PMC9617856; doi:10.1038/s41467-022-34012-2)
Supplement: Supplementary file 2 — Description of Additional Supplementary Files [file 41467_2022_34012_MOESM2_ESM.pdf]

Description of Additional Supplementary Files

File Name: Supplementary Data 1
Description: Per patient clinical information for Barrier (Sheet 1), CPC (Sheet 2), WCDT (Sheet 3) and VPC (Sheet 4) cohorts. Sample batch information (Sheet 5).

File Name: Supplementary Data 2
Description: Bin level raw count matrix for all samples.

File Name: Supplementary Data 3
Description: Normalized peak level intensity.

File Name: Supplementary Data 4
Description: Data source for public ChIP-seq data used in this study.
