# Supplementary material for: The cell-free DNA methylome captures distinctions between localized and metastatic prostate tumors
Source: Nat Commun. 2022 Oct 29;13:6467. doi: 10.1038/s41467-022-34012-2 (PMC9617856; doi:10.1038/s41467-022-34012-2)
Supplement: Supplementary file 7 — Reporting Summary [file 41467_2022_34012_MOESM7_ESM.pdf]

nature portfolio

Corresponding author(s): Housheng Hansen He, Alexander W. Wyatt, Felix Feng, Guoli Ji

Last updated by author(s): Oct 6, 2022

Reporting Summary

Nature Portfolio wishes to improve the reproducibility of the work that we publish. This form provides structure for consistency and transparency in reporting. For further information on Nature Portfolio policies, see our Editorial Policies and the Editorial Policy Checklist.

Statistics

For all statistical analyses, confirm that the following items are present in the figure legend, table legend, main text, or Methods section.

n/a	Confirmed	
☐	☒	The exact sample size (*n*) for each experimental group/condition, given as a discrete number and unit of measurement
☐	☒	A statement on whether measurements were taken from distinct samples or whether the same sample was measured repeatedly
☐	☒	The statistical test(s) used AND whether they are one- or two-sided<br>*Only common tests should be described solely by name; describe more complex techniques in the Methods section.*
☐	☒	A description of all covariates tested
☐	☒	A description of any assumptions or corrections, such as tests of normality and adjustment for multiple comparisons
☐	☒	A full description of the statistical parameters including central tendency (e.g. means) or other basic estimates (e.g. regression coefficient) AND variation (e.g. standard deviation) or associated estimates of uncertainty (e.g. confidence intervals)
☐	☒	For null hypothesis testing, the test statistic (e.g. F, t, r) with confidence intervals, effect sizes, degrees of freedom and P value noted<br>*Give P values as exact values whenever suitable.*
☒	☐	For Bayesian analysis, information on the choice of priors and Markov chain Monte Carlo settings
☒	☐	For hierarchical and complex designs, identification of the appropriate level for tests and full reporting of outcomes
☐	☒	Estimates of effect sizes (e.g. Cohen's *d*, Pearson's *r*), indicating how they were calculated

Our web collection on statistics for biologists contains articles on many of the points above.

Software and code

Policy information about availability of computer code

Data collection	*Provide a description of all commercial, open source and custom code used to collect the data in this study, specifying the version used OR state that no software was used.*
Data analysis	All data analysis details, including method and detailed parameters are described in Methods.<br>Software used: R statistical environment (v3.6.1); FastQC (v0.11.5); MultiQC (v0.8). BWA (v0.7.15); SAMtools (v1.3.1); Picard; MACS (v2.2.5); ucsctools (v378); bwtool (v1.0); CaSpER (v0.1.0), liftOver (v1.10.0).<br>R packages: sva(3.32.1); DESeq2 (v1.24.0); MEDIPS (v1.34.0); annotatr (v1.12.1); TxDb.Hsapiens.UCSC.hg19.knownGene (v3.2.2); org.Hs.eg.db (v3.10.0); GenomicRanges (v1.38.0); regioneR (v1.16.2); Gviz (v1.30.3); TCGAbiolinks (v2.14.0); CaSpER (v0.1.0); randomForest (v4.6.14); survminer (v0.4.6); picard (v2.6.0);ROCR (v1.07).<br>Customized script from previous study: http:github.com/Cancer-Genomics/delfi_scripts

For manuscripts utilizing custom algorithms or software that are central to the research but not yet described in published literature, software must be made available to editors and reviewers. We strongly encourage code deposition in a community repository (e.g. GitHub). See the Nature Portfolio guidelines for submitting code & software for further information.

Data

Policy information about availability of data

All manuscripts must include a data availability statement. This statement should provide the following information, where applicable:

- Accession codes, unique identifiers, or web links for publicly available datasets
- A description of any restrictions on data availability
- For clinical datasets or third party data, please ensure that the statement adheres to our policy

The raw cfMeDIP-seq data generated in this study have been deposited in the European Genome-Phenome Archive (EGA) database under the study accession code EGAS00001005522 (https://ega-archive.org/studies/EGAS00001005522) and the dataset accession codes EGAD00001007972, EGAD00001008711, EGAD00001008712, EGAD00001008713, EGAD00001008737 (https://ega-archive.org/datasets/EGAD00001007972, https://ega-archive.org/datasets/EGAD00001008711, https://ega-archive.org/datasets/EGAD00001008712, https://ega-archive.org/datasets/EGAD00001008713, https://ega-archive.org/datasets/EGAD00001008737). The raw data are available under restricted access due to them containing identifying information that could compromise patient privacy. Access can be obtained by contacting the data access committee listed on the EGA page and according to the EGA guideline. There are no restrictions on data access application. Applications will be reviewed monthly, and once all patient privacy and data transfer documents are completed; we will notify EGA within two-weeks to allow data downloading. Immediately upon receipt of our notification, EGA will create an account for the applicant to download data and the timeframe to download data will be in accordance with EGA guidance. The processed bin level raw count data are provided in Supplementary Data 2 on Open Science Framework (OFS, https://osf.io/97tqk/); normalized peak level intensity are available in Supplementary Data 3 on OFS (https://osf.io/97tqk/). Source data are provided with this paper.
The human hg19 reference genome and the chain file for liftOver was downloaded from the UCSC genome browser (https://hgdownload.soe.ucsc.edu/goldenPath/hg19/bigZips/genes/ and https://hgdownload.soe.ucsc.edu/goldenPath/hg38/liftOver/). The public panel gene analysis data for the VPC cohort used in this study are available in the EGA database under accession code EGAS00001003113 (https://ega-archive.org/studies/EGAS00001003113)23. The publicly available data from the CPC cohort used in this study are available in the EGA database under accession code EGAS00001000900 (https://ega-archive.org/studies/EGAS00001000900)20,22,24. The publicly available data from the CPGEA cohort used in this study are available in the National Genomics Data Center (NGDC) under the accession code PRJCA001124 (https://bigd.big.ac.cn/search?dbId=&q=PRJCA001124), and the processed data can be accessed at: http://www.cpgea.com 46. The publicly available data from the WCDT cohort used in this study are available in the database of Genotypes and Phenotypes (dbGAP) under the accession code phs001648 (https://www.omicsdi.org/dataset/dbgap/phs001648), and the processed data can be accessed at: http://davidquigley.com/prostate.html 21. The publicly available data TCGA data used in this study are available in the Broad Institute FireBrowse portal (http://firebrowse.org/?cohort=PRAD) 47. Three genes with promoter hypomethylation in prostate cancer were obtained from a previous report37. A list of 136 prostate cancer driver genes was obtained from previous publication38 and database DriverDBv339 (http://driverdb.tms.cmu.edu.tw/api/get_source_file?type=txt&cate=Cancer&symbol=250005866&tab=summary&file=summary_tab.txt). Source data are provided with this paper as a Source Data file. The remaining data are available within the Article, Supplementary Information or Source Data file.

Field-specific reporting

Please select the one below that is the best fit for your research. If you are not sure, read the appropriate sections before making your selection.

☒ Life sciences ☐ Behavioural & social sciences ☐ Ecological, evolutionary & environmental sciences

For a reference copy of the document with all sections, see nature.com/documents/nr-reporting-summary-flat.pdf

Life sciences study design

All studies must disclose on these points even when the disclosure is negative.

Sample size	As an exploratory study, no formal sample size calculation was performed. Blood samples in this study were obtained as part of existing cohort or clinical trials where other genomic profiling was performed, it is thus limited to the availability of remaining samples.
Data exclusions	Samples need to satisfy two steps of quality controls (QC) before proceeding to cfMeDIP-seq sequencing: QC1 was performed by qPCR to detect recovery of the spiked-in methylated and unmethylated A. thaliana DNA. The recovery of methylated A. thaliana DNA should be >20%, unmethylated A. thaliana DNA should be <1% (relative to the input control and adjusted to input control being 10% of the overall sample), and the specificity of the reaction should be >99% (1- [recovery of spike-in unmethylated DNA/ recovery of spike-in methylated DNA] x 100) to proceed. QC2 was determined by qPCR, where the cycle number for library amplification should be <15 cycles.
Replication	One replication per sample was obtained.
Randomization	The work requires no randomization: Human specimen were allocated into groups according to disease status (localized prostate cancer and mCRPC).
Blinding	Blinding is not relevant as no treatment for patient is needed.

Reporting for specific materials, systems and methods

We require information from authors about some types of materials, experimental systems and methods used in many studies. Here, indicate whether each material, system or method listed is relevant to your study. If you are not sure if a list item applies to your research, read the appropriate section before selecting a response.

Materials & experimental systems

n/a	Involved in the study
☐	☒ Antibodies
☒	☐ Eukaryotic cell lines
☒	☐ Palaeontology and archaeology
☒	☐ Animals and other organisms
☐	☒ Human research participants
☒	☐ Clinical data
☒	☐ Dual use research of concern

Methods

n/a	Involved in the study
☒	☐ ChIP-seq
☒	☐ Flow cytometry
☒	☐ MRI-based neuroimaging

Antibodies

Antibodies used

5mC monoclonal antibody (C15200081, Diagenode, clone 33D3)

Validation

5mC monoclonal antibody (C15200081, Diagenode) is validated by vendor by MeDIP (Seq and qPCR), dot blot, Immunofluorescence (IF), and Surface plasmon resonance (SPR) analysis against 5mC. Species reactivity for human is positive.
Detailed information can be found here: https://www.diagenode.com/en/p/5-mc-monoclonal-antibody-33d3-premium-100-ug-50-ul

Human research participants

Policy information about studies involving human research participants

Population characteristics

All patients involved in this study were male with prostate cancer. Patients with localized prostate cancer were subjected to prostatectomy. Patients with mCRPC were subjected to AR inhibition treatment.
All patients are male, age range 44-92, per patient age and clinical information can be find in Supplementary Data 1.

Recruitment

All samples were collected from existing cohort or clinical trial studies, sample availability serves as a limitation factor. The first batch of VPC samples were selected for samples with higher ctDNA load, while the second batch sequenced remaining ones, thus covering the full spectrum of available samples. There are no other criteria for selecting patients to this study. There are no self-selection bias or other biases in recruitment.

Ethics oversight

All patients provided informed consent and all samples were obtained upon approval of the institutional ethics committee and Research Ethics Board at the University Health Network (UHN) and the University of British Columbia (UBC), with compliance to all relevant ethical regulations. CPC and Barrier samples were retrieved from the UHN GU Biobank (REB file numbers: 11-0024(CPC) and 13-7122(Barrier)). VPC cfDNA from plasma was retrieved from the Vancouver Prostate Centre (VPC), UBC (REB file number: H18-00944).

Note that full information on the approval of the study protocol must also be provided in the manuscript.
